# Current practices in the diagnosis and management of HSV and CMV reactivation in German ICUs: an exploratory web-based survey

**DOI:** 10.3389/fmed.2026.1884844

**Published:** 2026-06-24

**Authors:** Stefanie Michel, Ivana Radisic, Stefan Hagel, Nina Pirschtat, Thorsten Brenner, Simon Dubler

**Affiliations:** 1 Department of Anaesthesiology and Intensive Care Medicine, University Hospital Essen, Essen, Germany; 2 Institute for Infectious Diseases and Infection Control, Jena University Hospital, Friedrich Schiller University, Jena, Germany

**Keywords:** antiviral therapy, cytomegalovirus (CMV), herpes simplex virus (HSV), intensive care medicine, viral diagnostics

## Abstract

**Background:**

Herpes simplex virus (HSV) and cytomegalovirus (CMV) reactivation are increasingly recognized in critically ill patients and may influence clinical outcomes. Diagnostic and therapeutic approaches vary considerably between institutions. We conducted an exploratory web-based survey to assess current practices regarding HSV and CMV reactivation in German intensive care units (ICUs).

**Methods:**

Between January 24 and March 5, 2025, 1,642 ICU directors in Germany were invited to complete an online questionnaire comprising 83 items addressing the diagnosis and treatment of HSV and CMV reactivation in critically ill patients, independent of immunosuppressive therapy.

**Results:**

The completion rate was 7.5%, with sufficient data for inclusion available from 123 questionnaires, of which 47 were fully completed and 76 were partially completed. Participating hospitals commonly had 501–1,000 beds and ICUs were primarily overseen by anaesthesiology departments with a median capacity of 14 beds. Standard operating procedures for HSV and CMV diagnostics were reported by 33% of institutions for transplant recipients and by 24% for non-immunocompromised ICU patients. According to respondents, virological testing was generally available several times per week, with a median turnaround time of 2 days. Profound heterogeneity in the reported diagnosis and management of HSV and CMV reactivation between surveyed ICUs was observed. Differences encompassed the reported implementation of routine testing, types of specimens and the diagnostic modalities applied, including serological and PCR-based approaches. Routine HSV and CMV testing was more frequently reported in immunosuppressed patients, particularly in solid organ and stem cell transplant recipients. In transplant recipients, PCR-based diagnostics and pre-emptive or prophylactic antiviral strategies were frequently employed, whereas in immunocompetent patients’ antiviral therapy was usually initiated only upon clinical suspicion, according to respondents.

**Conclusion:**

This exploratory survey among responding ICUs suggests substantial variability in reported diagnostic and therapeutic approaches to HSV and CMV reactivation in German critical care practice. The findings highlight current practice heterogeneity and areas of clinical uncertainty, particularly in critically ill patients without primary immunosuppression.

## 1 Introduction

Reactivation of human herpesviruses, particularly herpes simplex virus (HSV) and cytomegalovirus (CMV), is frequently observed in critically ill patients in intensive care units (ICUs) (1–3). Patients with sepsis are at especially high risk for viral reactivation (4), which has been associated with an unfavourable clinical course and poor outcomes (5–8). While the clinical significance and prognostic implications of herpesvirus reactivation have been extensively characterised in solid organ and haematopoietic stem cell transplant recipients as well as in other immunosuppressed patient populations, their relevance in primarily non-immunocompromised critically ill patients remains insufficiently understood (9, 10). Despite the increasing availability of virological diagnostic methods, clear recommendations regarding the indication, timing, and extent of viral diagnostics and antiviral therapy in this setting are still lacking (11).

Several factors, including disease severity, the need for respiratory support, the presence of concomitant infections, and pharmacological immunosuppression, may substantially influence both the risk of viral reactivation and its prognostic relevance (12, 13). It remains unclear whether herpesvirus reactivations in critically ill patients should primarily be interpreted as epiphenomena of severe systemic dysregulation or whether they independently contribute to clinical deterioration as a comorbid condition (14). HSV-1 reactivation in critically ill patients has been shown to occur frequently in blood and lower airways and was associated with increased mortality and higher rates of ventilator-associated pneumonia (15). At the same time, overinterpretation of viral detection as evidence of a primary pathogenic process may potentially misguide clinical decision-making and divert attention from the causative pathogen (16). Consequently, the clinical interpretation of HSV and CMV reactivation is complicated by a multitude of potentially confounding factors, and there is conflicting evidence regarding the benefit of antiviral therapy following viral detection (17–19).

A better understanding of current clinical practice in ICUs, including existing diagnostic and therapeutic strategies, is essential to characterise prevailing approaches, identify areas of uncertainty, and establish a foundation for future evidence-based recommendations. The aim of the present study was to systematically assess diagnostic and therapeutic approaches to HSV and CMV reactivation in German ICUs and to map the variability in clinical management across institutions.

## 2 Methods

### 2.1 Study design and setting

We conducted a multicentre, cross-sectional survey study to systematically assess the diagnostic and therapeutic management of HSV and CMV reactivation in German ICUs. The survey was conducted between January 24 and March 5, 2025. A total of 1,642 ICU lead physicians, all of whom were members of the German Interdisciplinary Association for Intensive Care and Emergency Medicine (Deutsche Interdisziplinäre Vereinigung für Intensiv- und Notfallmedizin, DIVI), were invited to participate. The survey was distributed online using personalised invitation links to prevent duplicate responses. No filter questions or adaptive branching were applied and all questions were voluntary. The estimated median time to complete the questionnaire was 20 min. Participation was anonymous. As this study was based exclusively on secondary, anonymised survey data and did not involve patient-level data, formal approval by an ethics committee was not required. The study was conducted in accordance with the principles of the Declaration of Helsinki. The full questionnaire is included as Supplementary material.

### 2.2 Survey instrument and data collection

A standardised questionnaire comprising 83 questions was developed to capture diagnostic and therapeutic strategies in the intensive care management of patients with HSV and/or CMV reactivation. The questionnaire included 71 single-choice questions, 4 questions allowing for multiple responses, and 8 open-ended (free-text only) responses. Eighteen questions addressed general structural characteristics and established institutional processes, while 65 questions focused on clinical diagnostic and therapeutic practices.

Data were collected anonymously using a web-based survey tool (LimeSurvey, Hamburg, Germany). The questionnaire captured demographic and professional characteristics of the participants, structural features of the respective ICUs, and current approaches to the diagnosis and treatment of HSV and CMV reactivation.

### 2.3 Outcomes

The primary outcome of the study was a descriptive characterisation of current diagnostic and therapeutic practices for HSV and CMV reactivation in German ICUs. Secondary outcomes aimed to identify structural differences in intensive care practice at the national level.

### 2.4 Statistical analysis

Analyses were performed using all available responses per item. Missing responses were handled on a per-question basis. The number of respondents may vary between questions and is reported as n in the figures and tables. A descriptive statistical analysis was performed. Categorical variables are presented as absolute and relative frequencies, and continuous variables as medians with interquartile ranges (IQRs). All analyses were conducted using established statistical software (RStudio, Posit, Boston, MA, USA; Microsoft™ Excel 365, Microsoft, Redmond, WA, USA).

## 3 Results

### 3.1 Structural characteristics of care

**Figure 1:**
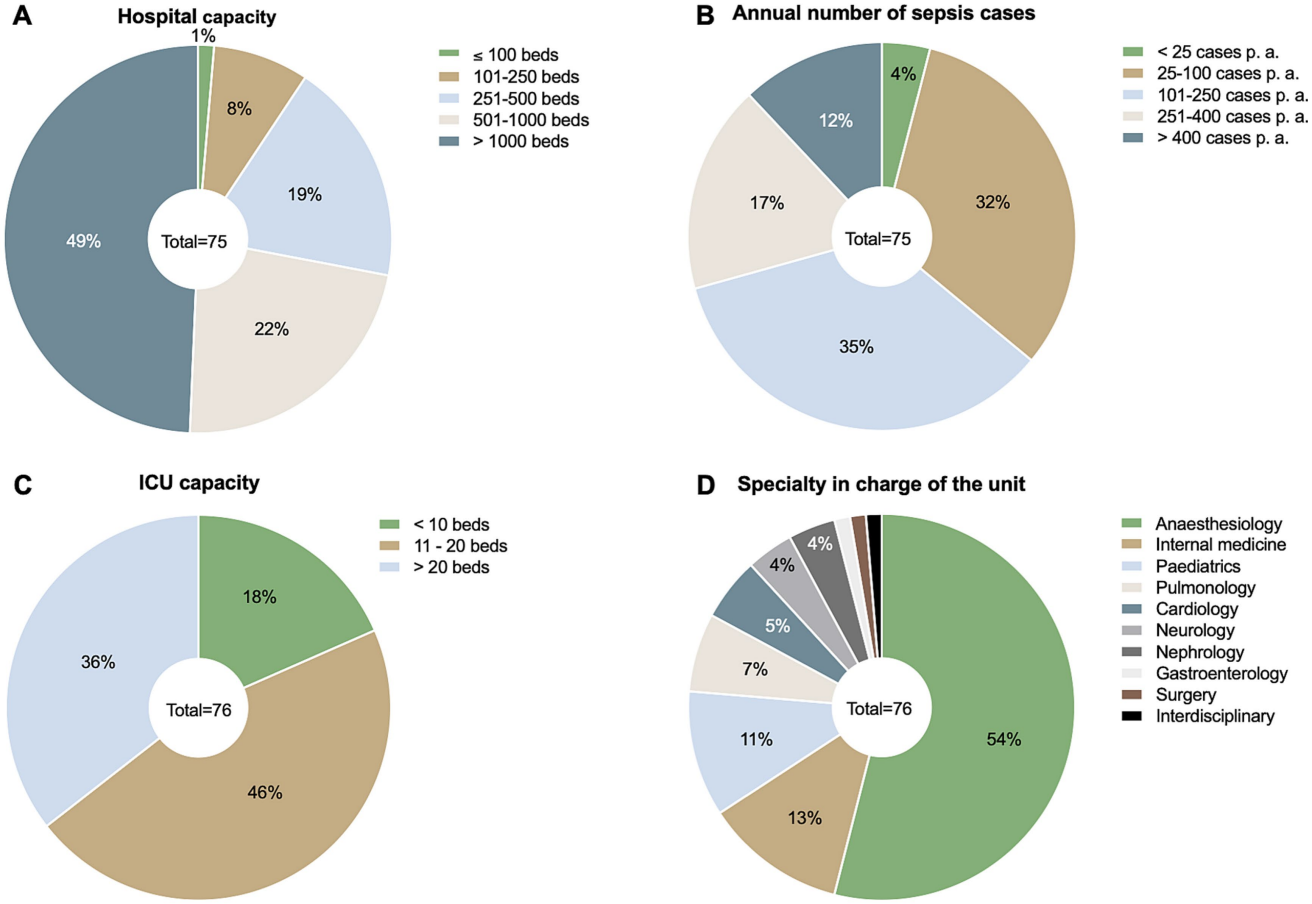
Participating institutions. Overview of participating institutions according to respondents’ reports: **(A)** hospital size, **(B)** number of sepsis cases per year, **(C)** ICU size, and **(D)** leading department responsible for the ICU.

Of 1,642 invited ICU physicians, 400 initiated the survey. Of these, 123 questionnaires provided sufficient data for inclusion in the final analysis, comprising 47 fully completed and 76 partially completed questionnaires. Thus, the initiation rate was 24.4%, while the proportion of invited physicians contributing analysable data was 7.5%. The lowest number of completed items among included questionnaires was 13. Most questionnaires were completed by senior physicians (consultants/attending physicians; 53%), followed by board-certified specialists (21%). Respondents predominantly worked at large hospitals with more than 1,000 beds (49%) and were mainly located in federal states such as Bavaria (21%) and North Rhine-Westphalia (20%). The participating ICUs had a median of 14 beds (interquartile range [IQR] 10–24) and were primarily led by departments of anaesthesiology (54%). Lead ICU disciplines were variable, with anaesthesiology being the most common lead discipline (54%), followed by internal medicine without subspecialisation (12%), paediatrics (11%), pulmonology (7%), cardiology (5%), and other disciplines (14%). The disciplines involved in ICU care irrespective of the leading role (multiple answers possible) were anaesthesiology (65%), gastroenterology (35%), cardiology (32%), surgery (29%), traumatology (28%), pulmonology (22%), and haematology/oncology (18%). According to respondents, the spectrum of treated patients was broad and mainly included gastroenterological (69%), visceral surgical (65%), orthopaedic (59%), cardiological (55%), neurological (51%), and neurosurgical cases (44%). Baseline characteristics are summarised in Figure 1.

All respondents reported sepsis as a common clinical condition, with an estimated average of 100–250 cases per ICU per year. The median proportion of invasively ventilated patients was 43% (IQR 27–60%), and the median ICU length of stay was 5 days (IQR 4–9). During routine clinical duty, one physician was responsible for a median of 8 patients (IQR 6–10), according to respondents. ICU routine medical staff included board-certified physicians in 39%, of whom 17% additionally held formal certification in intensive care medicine. Respondents reported a nurse-to-patient ratio of 2:1, and 47% of nurses were certified intensive care and anaesthesia nurses.

On-site virological diagnostics were available in 61% of the participating centres. Solid organ transplantation was performed in 42% of centres according to reports, autologous haematopoietic stem cell transplantation in 41%, and allogeneic stem cell transplantation in 37%. Overall, only 61% of ICUs reported regularly caring for transplant recipients (solid organ, autologous, or allogeneic stem cell transplantation).

### 3.2 Diagnostic strategies

**Figure 2:**
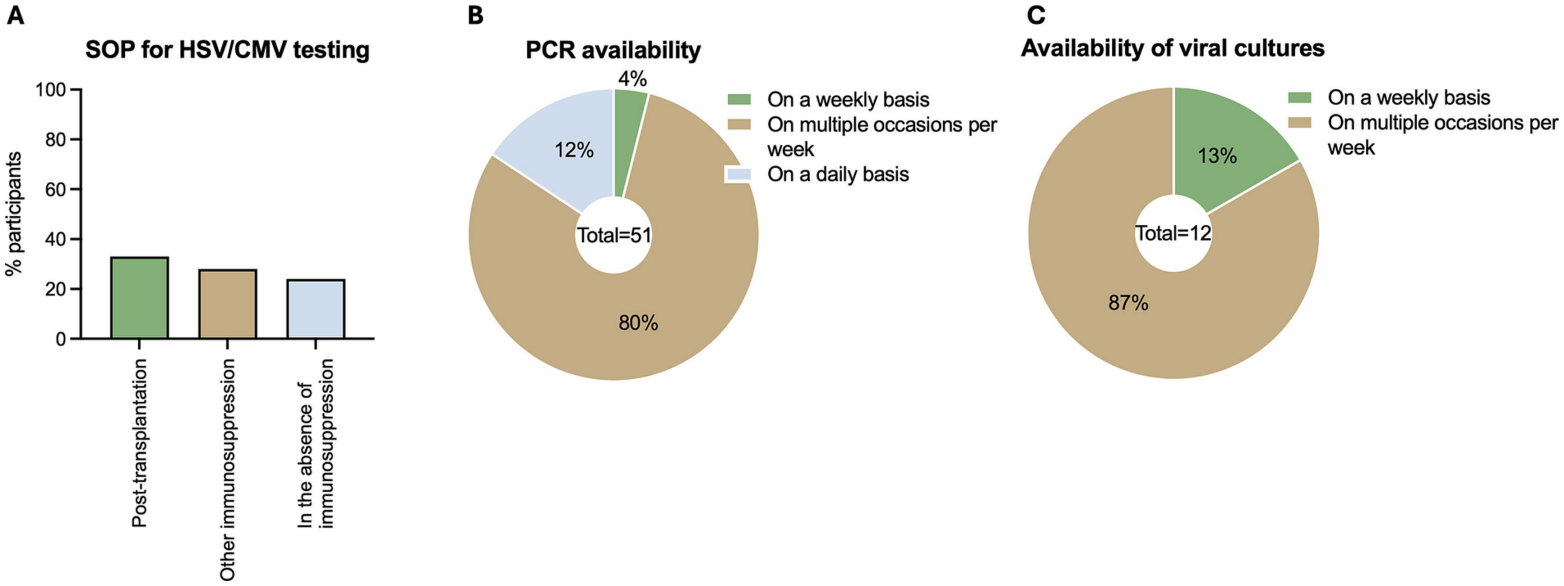
Structural prerequisites. Structural prerequisites reported by participating ICUs: **(A)** availability of a standard operating procedure (SOP), **(B)** availability of polymerase chain reaction (PCR)-based diagnostics, and **(C)** availability of viral culture for diagnostic purposes.

Written standard operating procedures (SOPs) for HSV and/or CMV diagnostics were reported with varying frequency depending on the clinical setting. SOPs for transplant recipients were available in 33% of ICUs, whereas SOPs for other immunosuppressed patients were reported by 28% of centres. SOPs applicable to ICU patients without immunosuppression were present in 24% of participating institutions (Figure 2).

Serological testing was more frequently reported for immunosuppressed patient populations than for non-immunosuppressed ICU patients. Following solid organ transplantation, serology was performed in 41% of cases for HSV and 44% for CMV; after autologous stem cell transplantation in 44% (HSV) and 50% (CMV); after allogeneic stem cell transplantation in 38% for both HSV and CMV; and in patients with other forms of immunosuppression in 49% (HSV) and 51% (CMV). In non-immunosuppressed ICU patients, routine serological testing was reported by only a minority of respondents (12% for HSV and 16% for CMV).

**Figure 3:**
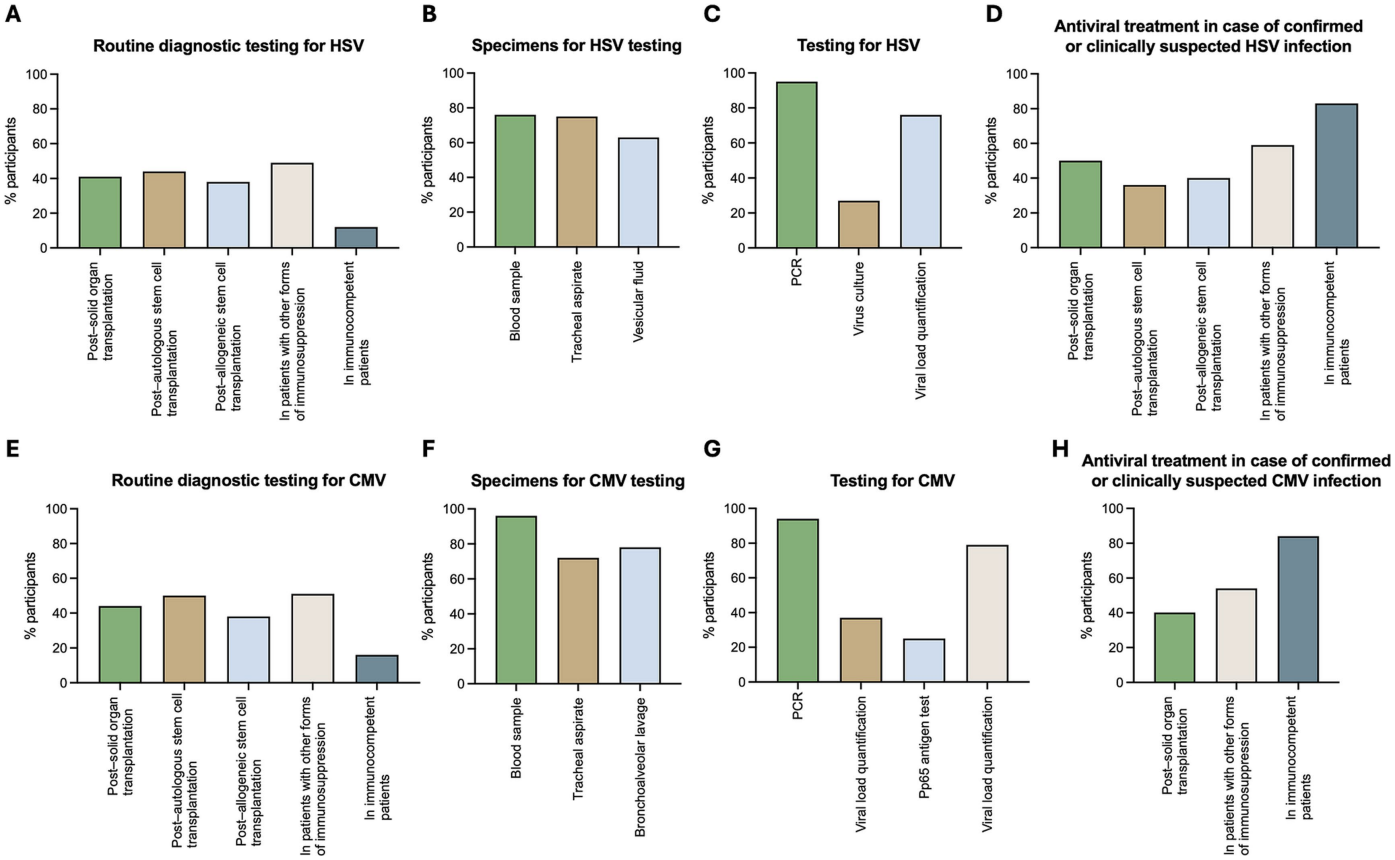
Viral diagnostics and antiviral therapy. Routine diagnostic strategies according to immunosuppression status, specimen type, diagnostic method, and antiviral treatment in the presence of viral detection, stratified by immunosuppression status for herpes simplex virus (HSV) **(A–D)** and cytomegalovirus (CMV) **(E–H)**, as reported by respondents.

PCR-based diagnostics were the preferred method when virological testing was indicated and were used primarily in high-risk populations. PCR testing was performed in nearly all allogeneic stem cell transplant recipients (94% for HSV and 100% for CMV), in autologous transplant recipients in 76% (HSV) and 86% (CMV), and in solid organ transplant recipients in 67% (HSV) and 83% (CMV). In patients with other forms of immunosuppression (e.g., immunosuppressive therapy or neutropenia), PCR was performed in 64% (HSV) and 65% (CMV), according to respondents. In non-immunosuppressed patients, PCR testing was applied in 29% (HSV) and 34% (CMV) of cases. The use of virological diagnostics is summarised in Figures 3A–C (HSV) and Figures 3E–G (CMV).

The most intensive CMV surveillance was reported for allogeneic stem cell transplant recipients, among whom 65% underwent repeated testing (>1 test per week) even in the absence of specific clinical suspicion. Corresponding proportions were 63% for autologous transplant recipients, 24% for solid organ transplant recipients, 33% for patients with other immunosuppression, and 11% for non-immunosuppressed ICU patients. Regular HSV screening was less common overall and reported as routinely performed in 38% of transplant recipients, 23% of patients with other immunosuppression, and 7% of non-immunosuppressed ICU patients.

In most centres (96%), virological testing could be performed multiple times per week if required. The median turnaround time for virological test results was 2 days (IQR 2–3). HSV diagnostics were most performed using blood serum (76%), tracheal secretions (75%), or vesicle fluid (63%). PCR was used in 95% of cases, whereas viral culture was performed in only 27%. Viral load quantification was reported in 76% of tests, with quantitative results provided in 95%; normalisation of viral load was performed in 57% of cases, according to respondents. For CMV, blood serum was the most frequently analysed specimen (96%), followed by bronchoalveolar lavage (BAL) samples (78%) and tracheal secretions (72%). Diagnostic testing was performed using PCR in 94% and serology in 82% of cases, while viral culture (37%) and antigen testing (pp65; 25%) were used less frequently. Quantitative viral load measurements were reported in 79% of tests. Reference thresholds were defined for 91% of blood samples but only for 64% of BAL samples.

### 3.3 Therapeutic strategies

For HSV, pre-emptive or prophylactic antiviral treatment strategies were more frequently reported for immunosuppressed patient populations. Pre-emptive therapy was reported in 34% of solid organ transplant recipients, 24% of autologous transplant recipients, 20% of allogeneic transplant recipients, and 34% of patients with other forms of immunosuppression. Prophylactic antiviral therapy was administered in 26, 40, 40, and 7% of these groups, respectively. For non-immunosuppressed patients, 83% of respondents reported initiating therapy only when HSV infection was clinically suspected or confirmed (Figure 3D).

A similar pattern was observed for CMV. Treatment thresholds were lower in high-risk populations such as solid organ transplant recipients, in whom antiviral therapy was initiated in the absence of overt clinical symptoms in 60% of cases. By contrast, 54% of respondents reported initiating treatment in patients with other forms of immunosuppression only when CMV infection was proven and considered clinically relevant, compared with 85% for non-immunosuppressed patients (Figure 3H).

## 4 Discussion

This exploratory nationwide survey of German ICUs indicated substantial variability in the diagnostic and therapeutic management of HSV and CMV reactivation in critically ill patients. Although virological testing was widely available, standardised operating procedures for viral diagnostics were present in only a minority of institutions, and diagnostic and therapeutic strategies differed considerably.

Current S2k guidelines on viral infections in solid organ and allogeneic stem cell transplant recipients recommend serological screening, virological monitoring, and prophylactic or pre-emptive antiviral therapy when indicated (20). Similarly, international consensus guidelines from the Transplantation Society emphasise the importance of systematic CMV management after solid organ transplantation (21). This is consistent with our survey data, demonstrating higher rates and closer intervals of viral testing in transplant recipients, paralleled by increased rates of antiviral therapy. Importantly, the comparatively low proportion of respondents reporting treatment only after confirmed or clinically suspected HSV or CMV infection should not be interpreted as a low overall treatment rate, as many centres instead reported prophylactic or pre-emptive treatment strategies. Nevertheless, the observed variation may reflect differences in institutional protocols, clinical treatment thresholds, and implementation of guideline recommendations. Comparable recommendations for the heterogenous population of critically ill, primarily non-immunosuppressed patients are lacking (17, 20). As reflected in our survey, this results in a wide range of individualised approaches that may depend on institutional size, clinical experience, and the availability of virological infrastructure, but may also reflect genuine clinical uncertainty. This uncertainty is understandable given the heterogeneous evidence base and the unresolved question of whether viral detection represents an epiphenomenon of severe critical illness, a marker of particularly vulnerable patients, or a clinically relevant viral infection requiring treatment. This ambiguity has important clinical implications. On the one hand, overinterpretation of viral detection may lead to unnecessary antiviral therapy, with potential adverse effects including nephrotoxicity, particularly in patients with impaired renal function or inadequate dose adjustment (22). Premature attribution of clinical deterioration to HSV or CMV reactivation may divert attention from alternative or concomitant causative pathogens. On the other hand, excessive diagnostic or therapeutic restraint may result in delayed recognition and undertreatment of clinically relevant viral reactivation with manifest organ disease.

Whether CMV and/or HSV reactivation in primarily non-immunosuppressed critically ill patients adversely affects clinical outcomes or merely represents a surrogate marker of severe underlying disease remains a matter of ongoing debate (14, 23). Several observational studies have reported frequent detection of HSV in ventilated ICU patients, some with an adverse impact on clinical outcomes and survival (13, 15, 19). In post-cardiac surgery patients, a mortality of up to 50% was reported, while the impact of HSV-1 activation on the primary cause of death remains uncertain (24). Semi-quantitative assessment of HSV-1 viral load in bronchoalveolar lavage has been proposed as one approach to improve clinical interpretation, as higher viral loads (e.g., >10^5^ copies/mL in bronchioalveolar lavage/bronchial aspirate) may be more suggestive of clinically relevant lower respiratory tract involvement (18, 25). HSV detection in bronchoalveolar lavage does not necessarily indicate HSV pneumonia, and it remains difficult to distinguish true HSV bronchopneumonitis from viral reactivation or colonisation in severely ill patients. Uncertainty regarding the interpretation and clinical relevance of test results may have contributed to the substantial heterogeneity in routine testing practices. Routine HSV testing was reported by only 38 to 49% of respondents for immunosuppressed patient populations, suggesting a lack of consensus, with practice almost evenly divided between centres that routinely tested and those that did not. In a routine diagnostic setting, almost half of HSV-positive BAL samples were accompanied by another typical respiratory virus or pathogenic microorganism as indicated by a recent study, thereby complicating attribution of respiratory disease to HSV and decisions regarding antiviral therapy. A study from the United States have questioned the routine inclusion of HSV PCR in bronchoalveolar lavage panels, as positive results often occurred together with other respiratory pathogens and rarely led to confirmed HSV pneumonia or antiviral treatment (16). Our data highlight that the greatest uncertainty concerns the management of non-immunosuppressed patients. Although 83% of respondents reported initiating antiviral therapy when clinically relevant HSV infection was suspected or confirmed, establishing the clinical significance of HSV detection in critically ill patients remains challenging and may substantially limit the practical clarity of this treatment criterion. The present findings therefore emphasise persistent uncertainty regarding when to perform testing, how to interpret positive results, and when antiviral treatment is warranted.

CMV reactivation is common in non-immunosuppressed critically ill patients and is associated with unfavourable clinical courses (23). However, causality and the benefit of antiviral therapy remain unclear, and routine treatment in primarily non-immunosuppressed patients is not recommended. A recent meta-analysis including 22 studies demonstrated an association between CMV reactivation and increased ICU mortality, prolonged mechanical ventilation, longer ICU length of stay, and higher rates of nosocomial infections in critically ill patients without primary immunosuppression (26). Conversely, a recent systematic review evaluating the impact of antiviral therapy on mortality in primarily immunocompetent patients with CMV reactivation failed to demonstrate a clear and consistent benefit regarding clinically relevant outcomes (27, 28). Similarly, a retrospective study in immunocompetent ICU patients identified CMV reactivation as a frequent finding associated with prolonged mechanical ventilation and longer hospital stay, but without a significant effect on mortality (5). The inconclusive and heterogeneous clinical evidence is mirrored by the survey findings. Routine CMV screening in immunosuppressed patients was reported by 38 to 51% of respondents, again indicating a lack of consensus, which was further reflected in the considerable variation in reported antiviral treatment strategies. Overall, interpretation in the context of incidental findings, viral load, concomitant pathogens, and the overall clinical trajectory may pose a major and unresolved clinical challenge.

Although the present survey was restricted to German ICUs, the observed uncertainty appears consistent with data from other healthcare systems. In French and Dutch cohorts of mechanically ventilated patients, pulmonary HSV and/or CMV reactivation was common, yet the distinction between viral bystander detection, marker of disease severity, and clinically relevant viral organ disease remained difficult (2, 14). The limited and partly conflicting interventional evidence further supports this uncertainty: a UK randomised trial showed that antiviral prophylaxis reduced CMV reactivation but did not establish a clear patient-centred benefit, while available studies and meta-analyses of acyclovir therapy for HSV detection in respiratory samples have not demonstrated a consistent survival advantage (10, 19). Thus, while our data reflect German ICU practice, they align with an international pattern of frequent viral detection, uncertain pathogenic relevance, and heterogeneous diagnostic and treatment decision-making.

The current findings underscore the need for a structured, risk-adapted approach to herpesvirus reactivation in the ICU setting beyond the dichotomous distinction between immunosuppressed and immunocompetent patient populations. Decisions regarding diagnostics and antiviral therapy should be guided by the overall clinical context, virological findings, organ involvement, and individual patient characteristics. In the future, improved identification of patient subgroups at particularly high risk may help refine diagnostic and therapeutic strategies for HSV and CMV reactivation in critically ill patients.

Prospective, randomised clinical trials are needed to systematically investigate the causal relationship between viral reactivation, virological parameters, therapeutic interventions, and patient-centred outcomes. In this context, the HerpMV trial initiated by Jena University Hospital (ClinicalTrials.gov Identifier: NCT06134492) represents a promising approach. This prospective, multicentre, open-label, randomised controlled two-arm trial evaluates whether antiviral therapy improves outcomes in critically ill patients with pneumonia and HSV-1 detection (≥10^3^ copies/mL in bronchoalveolar lavage). Patients are randomised 1:1 to receive either acyclovir (10 mg/kg every 8 h for 10 days or until ICU discharge) or no antiviral treatment. The primary endpoint is 30-day mortality, with secondary endpoints including ventilator-free and vasopressor-free days up to day 30 (27).

### 4.1 Limitations

The findings should be interpreted with caution given the limited completion rate. Of 1,642 invited ICU lead physicians, 400 initiated the survey and 123 questionnaires provided sufficient data for inclusion in the final analysis, which may have introduced selection bias. ICUs with greater interest in virological diagnostics, infectious complications, or transplant medicine may have been more likely to respond. Although low response rates [3.2, 1.2, and 2.5%, respectively (29–31)] for voluntary physician mail surveys are common, the results should not be considered fully representative of all German ICUs. Potential reasons for the low response rate include the length of the questionnaire, the voluntary web-based design, survey fatigue, time constraints among ICU physicians, and lower perceived relevance among centres with limited exposure to virological diagnostics or transplant medicine. Still, the included responses covered a broad range of ICU structures and clinical settings, allowing an exploratory assessment of current practice patterns and areas of uncertainty. Patient-level data including mortality, duration of mechanical ventilation, and nosocomial infections are necessary for direct conclusions regarding the impact of diagnostic or therapeutic strategies on clinical outcomes. Finally, the cross-sectional design does not allow for causal inference or assessment of temporal dynamics of viral reactivation and its clinical relevance.

## 5 Conclusion

The management of HSV and CMV reactivation in responding German ICUs was characterised by substantial variability in diagnostic and therapeutic approaches. These findings highlight current practice heterogeneity and areas of clinical uncertainty, particularly in critically ill patients without primary immunosuppression. Future prospective, multicentre studies are needed to clarify the prognostic relevance of viral reactivation, evaluate the potential benefit and risks of antiviral strategies, and support the development of risk-adapted diagnostic and therapeutic concepts.

## Data Availability

The raw data supporting the conclusions of this article will be made available by the authors, without undue reservation.
