# Supplementary material for: Current practices in the diagnosis and management of HSV and CMV reactivation in German ICUs: an exploratory web-based survey
Source: Front Med (Lausanne). 2026 Jun 24;13:1884844. doi: 10.3389/fmed.2026.1884844 (PMC13342238; doi:10.3389/fmed.2026.1884844)
Supplement: Supplementary file 1 [file Data_Sheet_1.PDF]

Supplementary material

Questionnaire (translation)

Current practices in the diagnosis and management of HSV and CMV reactivation in German ICUs: an exploratory web-based survey

What is your professional qualification?

- Resident physician
- Board-certified specialist
- Senior physician
- Lead physician
- Head of department

In which federal state is your hospital located?

- Baden-Württemberg
- Bavaria
- Berlin
- Brandenburg
- Bremen
- Hamburg
- Hesse
- Mecklenburg-Western Pomerania
- Lower Saxony
- North Rhine-Westphalia
- Rhineland-Palatinate
- Saarland
- Saxony
- Saxony-Anhalt
- Schleswig-Holstein
- Thuringia

How many hospital beds does your hospital have in total?

- <100
- 100-250

- 251-500
- 501-1,000
- >1,000

How many intensive care beds (beds equipped for invasive mechanical ventilation; no intermediate care units) is your department responsible for?

- Free-text response

Which specialty has primary responsibility for the management of your ICU?

- Anaesthesiology
- General and visceral surgery
- Neurosurgery
- Trauma surgery / Orthopaedics
- Cardiac surgery
- Pulmonology
- Cardiology
- Gastroenterology and hepatology
- Haematology and oncology
- Paediatrics
- Neurology

Patients from which specialties are treated on your ICU?

- Anaesthesiology
- General and visceral surgery
- Neurosurgery
- Trauma surgery / Orthopaedics
- Cardiac surgery
- Pulmonology
- Cardiology
- Gastroenterology and hepatology
- Haematology and oncology
- Paediatrics
- Neurology

How many patients with sepsis or septic shock are treated annually in your department?

- <25
- 25-100

- 101-250
- 251-400
- >401

Which medical specialties are represented in your ICU medical staff?

- Anaesthesiology
- General and visceral surgery
- Neurosurgery
- Trauma surgery / Orthopaedics
- Cardiac surgery
- Pulmonology
- Cardiology
- Gastroenterology and hepatology
- Haematology and oncology
- Paediatrics
- Neurology

What proportion of patients on your ICU receive invasive mechanical ventilation?

- Free-text response

What is the physician-to-patient staffing ratio on your ICU during off-hours (after 4:00 PM or weekends)?

- Free-text response

What proportion of physicians in your ICU team are board-certified specialists?

- Free-text response

What is the average length of stay on your ICU?

- Free-text response

What is the nurse-to-patient staffing ratio on your ICU?

- Free-text response

How many physicians hold the additional qualification in “specialised intensive care medicine”?

- Free-text response

What proportion of your nursing staff has specialised ICU nursing training?

- Free-text response

Where is the virology department located?

- Within the same hospital (“in-house”)
- Within the same hospital network

Are transplant procedures performed at your institution?

- Solid organ transplantation
- Autologous stem cell transplantation
- Allogeneic stem cell transplantation

Are transplanted patients regularly treated on your ICU?

- Yes
- No

In patients after solid organ transplant: do you regularly conduct serological HSV testing?

- Yes
- No

In patients after solid organ transplant: do you regularly conduct serological CMV testing?

- Yes
- No

In patients after autologous stem cell transplantation: do you routinely perform serological HSV testing?

- Yes

- No

In patients after autologous stem cell transplantation: do you routinely perform serological CMV testing?

- Yes
- No

In patients after allogeneic stem cell transplantation: do you routinely perform serological HSV testing?

- Yes
- No

In patients after allogeneic stem cell transplantation: do you routinely perform serological CMV testing?

- Yes
- No

In other immunocompromised patients (e.g., due to medication for autoimmune diseases or neutropenia), do you routinely perform serological HSV testing?

- Yes
- No

In other immunocompromised patients (e.g., due to medication for autoimmune diseases or neutropenia), do you routinely perform serological CMV testing?

- Yes
- No

In intensive care patients without immunosuppression, do you routinely perform serological HSV testing?

- Yes
- No

In intensive care patients without immunosuppression, do you routinely perform serological CMV testing?

- Yes
- No

In patients after solid organ transplantation: do you routinely perform HSV testing by PCR?

- Yes
- No

In patients after solid organ transplantation: do you routinely perform CMV testing by PCR?

- Yes
- No

In patients after autologous stem cell transplantation: do you routinely perform HSV testing by PCR?

- Yes
- No

In patients after autologous stem cell transplantation: do you routinely perform CMV testing by PCR?

- Yes
- No

In patients after allogeneic stem cell transplantation: do you routinely perform HSV testing by PCR?

- Yes
- No

In patients after allogeneic stem cell transplantation: do you routinely perform CMV testing by PCR?

- Yes
- No

In other immunocompromised patients (e.g., due to medication for autoimmune diseases or neutropenia), do you routinely perform HSV testing by PCR?

- Yes
- No

In other immunocompromised patients (e.g., due to medication for autoimmune diseases or neutropenia), do you routinely perform CMV testing by PCR?

- Yes
- No

In intensive care patients without immunosuppression, do you routinely perform HSV testing by PCR?

- Yes
- No

In intensive care patients without immunosuppression, do you routinely perform CMV testing by PCR?

- Yes
- No

At what frequency is CMV testing performed in patients after solid organ transplantation?

- Targeted testing based on clinical suspicion
- Weekly
- More frequently
- Less frequently
- Not performed (no transplant patients)

How often is CMV testing performed in patients after allogeneic stem cell transplantation?

- Targeted testing based on clinical suspicion
- Weekly
- More frequently
- Less frequently
- Not performed (no transplant patients)

How often is CMV testing performed in patients after autologous stem cell transplantation?

- Targeted testing based on clinical suspicion
- Weekly
- More frequently
- Less frequently
- Not performed (no transplant patients)

How often is CMV testing performed in other immunosuppressed patients (medication or neutropenia)?

- Targeted testing based on clinical suspicion
- Weekly
- More frequently
- Less frequently
- Not performed (no immunosuppressed patients)

How frequently is CMV testing performed in non-immunocompromised patients?

- Targeted testing based on clinical suspicion
- Weekly
- More frequently
- Less frequently

How frequently is HSV testing performed in transplant patients?

- Targeted testing based on clinical suspicion
- Weekly
- More frequently
- Less frequently
- Not performed (no transplant patients)

How frequently is HSV testing performed in other immunocompromised patients (e.g., due to medication or neutropenia)?

- Targeted testing based on clinical suspicion
- Weekly
- More frequently
- Less frequently
- Not performed (no immunocompromised patients)

How frequently is HSV testing performed in non-immunocompromised patients?

- Targeted testing based on clinical suspicion
- Weekly
- More frequently
- Less frequently

Is there a written protocol for viral diagnostics in transplant patients?

- Yes
- No

Is there a written protocol for viral diagnostics in other immunocompromised patients (e.g., due to medication or neutropenia)?

- Yes
- No

Is there a written protocol for viral diagnostics in ICU patients without immunosuppression?

- Yes
- No

Which specimen types and methods are used for HSV testing: vesicle fluid?

- Yes
- No

Which specimen types and methods are used for HSV testing: tracheal secretions?

- Yes
- No

Which specimen types and methods are used for HSV testing: bronchoalveolar lavage (BAL)?

- Yes
- No

Which specimen types and methods are used for HSV testing: serology?

- Yes

- No

Which specimen types and methods are used for HSV testing: PCR?

- Yes
- No

Which specimen types and methods are used for HSV testing: viral culture?

- Yes
- No

Is a quantitative HSV viral load reported?

- Yes
- No

If yes, how are results reported?

- Categorical (positive, weakly positive, negative)
- Quantitative (copies/mL or CT value)
- Semi-quantitative (estimated from PCR CT curve using an internal standard)

Serological HSV diagnostics: what is the average turnaround time for results?

- 1 day
- 2 days
- 3 days
- 4 days
- 5 days

HSV PCR diagnostics: when and how often are the respective testing methods available to you?

- On request only
- Once weekly
- Twice weekly
- Three times weekly
- Four times weekly
- Daily (excluding weekends)

- Daily (including weekends)

HSV viral culture: when and how frequently are the respective testing methods available to you?

- On request only
- Once weekly
- Twice weekly
- Three times weekly
- Four times weekly
- Daily (excluding weekends)
- Daily (including weekends)

Is the reported quantitative viral load normalised (e.g., normalised to a housekeeping gene)?

- Yes
- No

Which specimen types and methods are used for CMV testing: blood?

- Yes
- No

Which specimen types and methods are used for CMV testing: tracheal secretions?

- Yes
- No

Which specimen types and methods are used for CMV testing: bronchoalveolar lavage (BAL)?

- Yes
- No

Which specimen types and methods are used for CMV testing: serology?

- Yes
- No

Which specimen types and methods are used for CMV testing: PCR?

- Yes
- No

Which specimen types and methods are used for CMV testing: viral culture?

- Yes
- No

Which specimen types and methods are used for CMV testing: antigenaemia test (pp65)?

- Yes
- No

Is a quantitative CMV viral load reported?

- Yes
- No

Serological CMV diagnostics: when and how frequently are the respective testing methods available to you?

- On request only
- Once weekly
- Twice weekly
- Three times weekly
- Four times weekly
- Daily (excluding weekends)
- Daily (including weekends)

CMV viral culture: when and how frequently are the respective testing methods available to you?

- On request only
- Once weekly
- Twice weekly
- Three times weekly
- Four times weekly
- Daily (excluding weekends)
- Daily (including weekends)

CMV PCR diagnostics: when and how frequently are the respective testing methods available to you?

- On request only
- Once weekly
- Twice weekly
- Three times weekly
- Four times weekly
- Daily (excluding weekends)
- Daily (including weekends)

CMV antigenaemia test (pp65): when and how frequently are the respective testing methods available to you?

- On request only
- Once weekly
- Twice weekly
- Three times weekly
- Four times weekly
- Daily (excluding weekends)
- Daily (including weekends)

Is the reported quantitative viral load normalized (blood: copies/ml or IU)?

- Yes
- No

Is the reported quantitative viral load normalized (BAL: positive, negative, or weakly positive)?

- Yes
- No

How do you determine the indication for antiviral therapy against HSV in transplant patients?

- Prophylactic (without viral detection in all transplant patients)
- Prophylactic (only in transplant patients at increased risk, e.g., HSV IgG-negative recipient from an IgG-positive donor)
- Pre-emptive (positive viral detection above a quantitative PCR threshold or positive viral culture, but still asymptomatic)
- Therapeutic only (only upon viral detection and clinical signs of viral organ disease e.g., pneumonia without other cause)

How do you determine the indication for antiviral therapy against HSV in patients after autologous stem cell transplantation?

- Prophylactic (without viral detection in all transplant patients)
- Prophylactic (only in transplant patients at increased risk, e.g., HSV IgG-negative recipient from an IgG-positive donor)
- Pre-emptive (positive viral detection above a quantitative PCR threshold or positive viral culture, but still asymptomatic)
- Therapeutic only (only upon viral detection and clinical signs of viral organ disease e.g., pneumonia without other cause)

How do you determine the indication for antiviral therapy against HSV in patients after allogeneic stem cell transplantation?

- Prophylactic (no viral detection in all transplant patients)
- Prophylactic in high-risk patients only
- Pre-emptive (positive viral detection without clinical symptoms)
- Targeted therapy only (viral detection plus clinical signs of viral organ disease)

How do you determine the indication for antiviral therapy against HSV in other immunocompromised patients (due to medication or neutropenia)?

- Prophylactic (no viral detection)
- Pre-emptive (positive viral detection without clinical symptoms)
- Targeted therapy only (viral detection plus clinical signs of viral organ disease)

How do you determine the indication for antiviral therapy against HSV in non-immunocompromised ICU patients?

- Prophylactic (no viral detection, in defined subgroups e.g., in sepsis/septic shock, ARDS or during prolonged ICU stay)
- Pre-emptive (positive viral detection without clinical symptoms)
- Targeted therapy only (viral detection plus clinical signs of viral organ disease)

How do you determine the indication for antiviral therapy against CMV in transplant patients?

- Prophylactic (without viral detection in all transplant patients)
- Prophylactic (only in transplant patients at increased risk e.g., CMV IgG-negative recipient from an IgG-positive donor)
- Pre-emptive (positive PCR, viral culture, or antigenaemia without symptoms)
- Targeted therapy only (viral detection plus clinical organ disease)

How do you determine the indication for antiviral therapy against CMV in patients who are immunosuppressed due to other medications or neutropenia?

- Prophylactic (without viral detection in all patients of this group)
- Pre-emptive (positive PCR, viral culture, or antigenaemia without symptoms)
- Targeted therapy only (viral detection plus clinically apparent organ manifestation)

How do you determine the indication for antiviral therapy against CMV in immunocompetent patients?

- Prophylactic (without viral detection in all patients of this group)
- Pre-emptive (positive PCR, viral culture, or antigenaemia without symptoms)
- Targeted therapy only (viral detection plus clinically apparent organ manifestation)
